# Vocal markers of schizophrenia: assessing the generalizability of machine learning models and their clinical applicability

**DOI:** 10.1192/j.eurpsy.2023.444

**Published:** 2023-07-19

**Authors:** A. Parola, A. Rybner, E. T. Jessen, M. Damsgaard Mortensen, S. Nyhus Larsen, A. Simonsen, Y. Zhou, K. Koelkebeck, V. Bliksted, R. Fusaroli

**Affiliations:** ^1^Aarhus University, Aarhus, Denmark; ^2^Chinese Academy of Sciences, Beijing, China; ^3^Hospital and Institute of the University of Duisburg-Essen, Essen, Germany

## Abstract

**Introduction:**

Machine learning (ML) approaches are a promising venue for identifying vocal markers of neuropsychiatric disorders, such as schizophrenia. While recent studies have shown that voice-based ML models can reliably predict diagnosis and clinical symptoms of schizophrenia, it is unclear to what extent such ML markers generalize to new speech samples collected using a different task or in a different language: the assessment of generalization performance is however crucial for testing their clinical applicability.

**Objectives:**

In this research, we systematically assessed the generalizability of ML models across contexts and languages relying on a large cross-linguistic dataset of audio recordings of patients with schizophrenia and controls.

**Methods:**

We trained ML models of vocal markers of schizophrenia on a large cross-linguistic dataset of audio recordings of 231 patients with schizophrenia and 238 matched controls (>4.000 recordings in Danish, German, Mandarin and Japanese). We developed a rigorous pipeline to minimize overfitting, including cross-validated training set and Mixture of Experts (MoE) models. We tested the generalizability of the ML models on: (i) different participants, speaking the same language (hold-out test set); (ii) different participants, speaking a different language. Finally, we compared the predictive performance of: (i) models trained on a single language (e.g., Danish) (ii) MoE models, i.e., ensemble of models (experts) trained on a single language whose predictions are combined using a weighted sum (iii) multi-language models trained on multiple languages (e.g., Danish and German).

**Results:**

Model performance was comparable to state-of-the art findings (F1: 70%-80%) when trained and tested on participants speaking the same language (out-of-sample performance). Crucially, however, the ML models did not generalize well - showing a substantial decrease of performance (close to chance) - when trained in a language and tested on new languages (e.g., trained on Danish and tested on German). MoE and multi-language models showed a better increase of performance (F1: 55%-60%), but still far from those requested for achieving clinical applicability.

**Conclusions:**

Our results show that the cross-linguistic generalizability of ML models of vocal markers of schizophrenia is very limited. This is an issue if our first goal is to translate these vocal markers into effective clinical applications. We argue that more emphasis needs to be placed on collecting large open datasets to test the generalizability of voice-based ML models, for example, across different speech tasks or across the heterogeneous clinical profiles that characterize schizophrenia spectrum disorder.

**Disclosure of Interest:**

None Declared

